# Influencing factors of emotional well-being in Chinese patients with gastroenteric tumours: the role of mindfulness practice, social support and patient-centred communication

**DOI:** 10.3389/fpsyt.2025.1550777

**Published:** 2025-11-17

**Authors:** Min Peng, Shu-Rui Liu, Shi-Yun Liu, Ruo-Xi Li, Yin-Ping Zhang

**Affiliations:** 1 School of Nursing, Health Science Center, Xi’an Jiaotong University, Xi’an, China; 2 Department of Nursing, Lanzhou Petrochemical General Hospital (The Fourth Affiliated Hospital of Gansu University of Chinese Medicine), Lanzhou, China; 3 School of Journalism and New Media, Xi’an Jiaotong University, Xi’an, China; 4 Faculty of Biology, Medicine and Health, The University of Manchester, Manchester, United Kingdom

**Keywords:** neoplasms, mental health, structural equation modelling, social support, cancer

## Abstract

**Objective:**

This study aimed to explore the factors influencing the emotional well-being of patients with gastroenteric tumours and to examine the mediating roles of beliefs about cancer and health self-efficacy in mindfulness practice, social support and patient-centred communication (PCC).

**Methods:**

A convenience sample of 517 patients with gastroenteric tumours who visited the oncology department of six tertiary hospitals in Lanzhou City, Gansu Province, between 26 July 2022 and 30 September 2022 was selected for this study. General demographic and disease information of the patients was collected, and the relationships among mindfulness practice, social support, PCC, beliefs about cancer, health self-efficacy and emotional well-being were analysed.

**Results:**

Mindfulness practice, social support, beliefs about cancer and health self-efficacy had positive effects on emotional well-being (*p* < 0.05). Beliefs about cancer mediated the relationships between mindfulness practice (effect size, 0.025), social support (effect size, 0.033), and PCC (effect size, 0.079) and emotional well-being. Notably, health self-efficacy also served as a mediator in the relationships between mindfulness practice (effect size, 0.093), social support (effect size, 0.040), PCC (effect size, 0.057) and emotional well-being, with all these mediation effects reaching statistical significance (*p* < 0.05).

**Conclusion:**

Social support, beliefs about cancer, mindfulness practice and health self-efficacy had positive effects on the emotional well-being of patients with gastroenteric tumours. Beliefs about cancer and health self-efficacy mediated the effects of mindfulness practice, social support and PCC on emotional well-being.

## 1 Introduction

In 2018, approximately 4.8 million people worldwide were newly diagnosed with gastrointestinal cancer, accounting for 26.3% of the global cancer incidence (1). Colorectal cancer accounted for 10.2%, gastric cancer 5.7%, liver cancer 4.7%, oesophageal cancer 3.2% and pancreatic cancer 2.5%, accounting for 35.4% of global cancer-related deaths (1). Gastrointestinal cancer poses a threat to the psychological well-being of patients, and patients with this disease and their families experience various physiological and psychological challenges during treatment, particularly anxiety and depression (2). The emotional well-being of patients with gastrointestinal cancer is associated with factors such as emotional support, health self-efficacy, beliefs about cancer and social support. Poor mental health not only hinders cancer treatment and recovery but also reduces patients’ quality of life and may even lead to suicidal behaviour (3–5).

Social support refers to the assistance and emotional or material support provided to patients by family, relatives, friends, colleagues or unions and organisations (6). Studies have shown that social support can not only provide practical care for patients with cancer but also offer psychological help, allowing them to cope positively with their illness (7). Beliefs about cancer, such as the conviction that treatment is futile or that death is inevitable, are often fatalistic, leading patients to refuse or be noncompliant with treatment. Patients holding these views may believe that treatment cannot change the inevitable outcome of death, which greatly impacts their psychological well-being by fostering a sense of hopelessness and despair (8). Conversely, holding more optimistic or adaptive beliefs about the potential benefits of treatment and the possibility of a better outcome, even if uncertain, may foster resilience. Health self-efficacy is an important psychological adaptive capacity for individuals facing the significant impact of a cancer diagnosis, influencing their confidence in managing symptoms, adhering to treatment and navigating life changes (9). Patient-centred communication (PCC) theory refers to a series of communication behaviours in the medical service process that aim to build trust, share information and make decisions collaboratively, thereby empowering patients (10). Mindfulness practice involves purposefully being aware of one’s current physical condition, thoughts, emotions and environment, expressing a non-judgmental and positive attitude towards them and simply acknowledging their existence without attempting to eliminate them (11). Previous research has primarily focused on the psychological anxiety of individual patients with cancer. Limited studies are available on mindfulness practice and PCC, and there are inconsistent findings regarding the effects of various influencing factors on emotional well-being (7, 8). It is imperative to pinpoint the factors that influence emotional well-being in patients who have survived cancer and comprehensively grasp the pathways through which these factors impact their emotional state. Specifically, understanding the nuances of which cancer beliefs are most detrimental or beneficial, and how they interact with other resources, such as social support and self-efficacy, is crucial for developing targeted interventions.

In Chinese culture, the concept of ‘filial piety’ is deeply ingrained. Many patients, particularly those who are middle-aged or elderly, often bear immense psychological pressure due to concerns about becoming a burden to their families. This cultural expectation of ‘considering the family first’ can sometimes lead patients to hide their condition or refuse necessary treatment, potentially harming their emotional state. Simultaneously, the family plays a central role in Chinese society. The emotional support and daily care provided by family members, especially spouses and children, often constitute the primary source of strength for patients in coping with the disease. This tight-knit family connection means that the social support network may play a more crucial role in patients’ emotional well-being than in other cultural contexts. Furthermore, in terms of doctor–patient communication, traditional notions of ‘respecting one’s teacher’ may lead patients to be more inclined to passively accept doctors’ recommendations rather than actively participating in discussions about their care. Therefore, examining how factors such as mindfulness practice, social support and PCC influence emotional well-being in patients who have survived cancer and comprehensively grasping the pathways through which these factors impact their emotional state holds significant theoretical importance. This exploration is also of practical value for developing intervention strategies tailored to the characteristics of Chinese culture.

**Figure 1:**
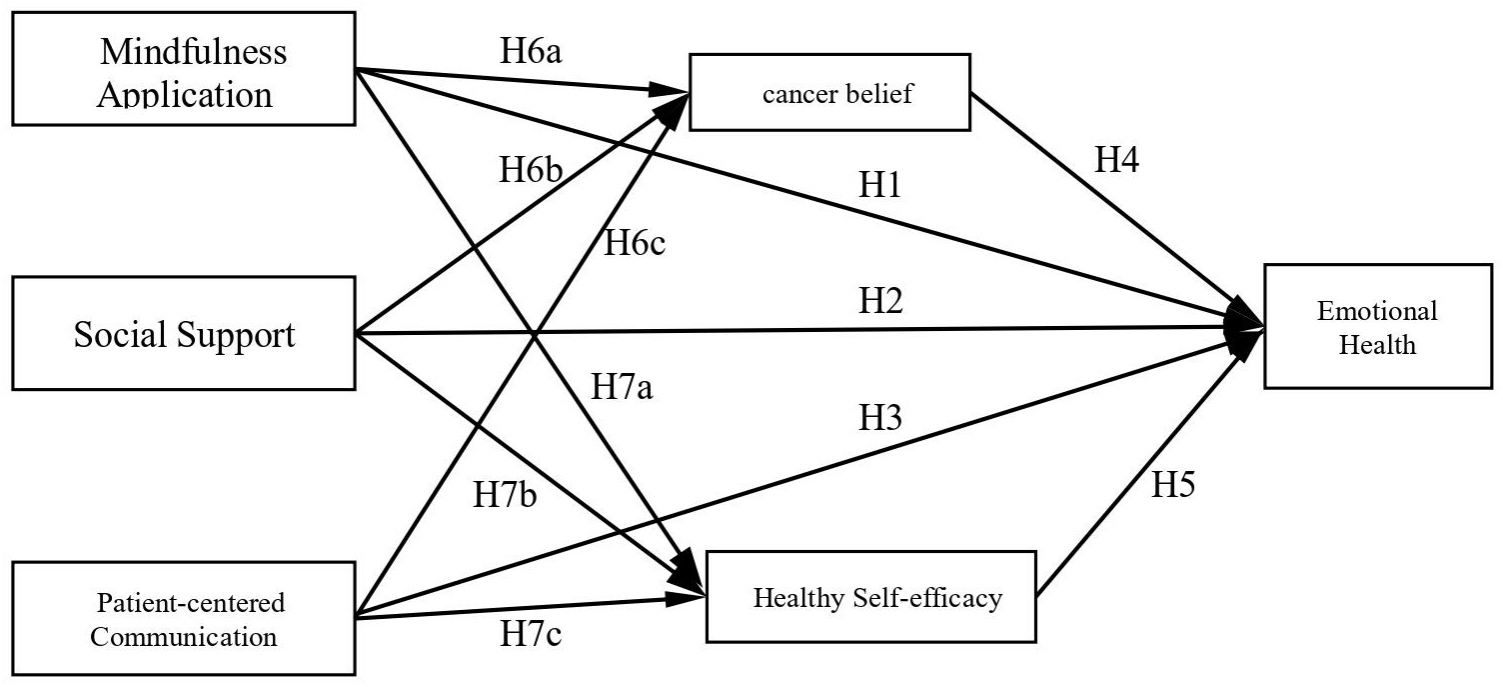
The research hypothesis diagram.

Additionally, beliefs about cancer and health self-efficacy may act as mediators in this relationship. Therefore, this study hypothesises the following: (1) there is a varying degree of correlation between mindfulness practice, social support, PCC and emotional well-being; (2) there is a correlation between mindfulness practice, social support, PCC and beliefs about cancer and health self-efficacy; (3) beliefs about cancer partially mediate the effects of mindfulness practice, social support and PCC on psychological well-being. This research’s hypothesis diagram is shown in **Figure 1**. This study aims to validate these hypotheses, quantify the contribution of the factors influencing emotional well-being and provide a scientific basis for targeted interventions of patients with cancer.

## 2 Materials and methods

### 2.1 Study participants

A total of 517 patients with gastrointestinal cancer who visited the oncology departments of six tertiary hospitals in Lanzhou City, Gansu Province – namely the Gansu Provincial Cancer Hospital, the First Affiliated Hospital of Lanzhou University, the Second Affiliated Hospital of Lanzhou University, the 904 Hospital of the Joint Service Support Force of the People’s Liberation Army of China, the First Affiliated Hospital of Gansu University of Chinese Medicine and the Gansu Gem Flower Hospital – were selected between 26 July 2022 and 30 September 2022. The inclusion criteria were as follows: (1) patients diagnosed accurately and consistently with gastrointestinal tumours through pathological examination; (2) aged ≥18 years (to exclude the interference of special physiological and psychological factors due to young age); (3) clear consciousness, basic communication and comprehension skills and ability to complete the relevant questionnaires and interviews normally; the validity of the research data collection was checked. The exclusion criteria were as follows: (1) patients with severe cognitive impairments, such that they could not accurately understand the research intentions and cooperate in completing the survey tasks, thus compromising the data quality; (2) other severe life-threatening diseases (such as concurrent advanced heart and lung failure or terminal malignant tumours in other parts); such diseases may cause complex physiological and psychological changes that interfere with the research results on the emotional health of patients with gastrointestinal tumours alone.

### 2.2 Survey method

This study employed a face-to-face paper-based survey to collect data. The questionnaire consisted of two main sections: general demographic information and emotional health-influencing factors. The section on emotional health included six dimensions (mindfulness practice, social support, PCC, cancer beliefs, health self-efficacy and emotional health) with a total of 30 items. All scales were based on mature or adapted authoritative instruments to ensure scientific validity and reliability. Prior to the survey, all researchers received unified training. The participants were instructed to complete the questionnaire independently; those with difficulties were assisted in accordance with the principle of neutral guidance without answer induction. After completion, researchers immediately checked the questionnaires for completeness and logical consistency. Questionnaires with more than three missing items or obviously patterned responses (e.g. consecutive identical choices) were reviewed and confirmed or supplemented by the participants to ensure data quality.

This study was approved by the Ethics Committee of the School of Nursing, Health Science Center of Xi’an Jiaotong University and adhered strictly to the principles of the Helsinki Declaration. All participants provided written informed consent after being fully informed of the research purpose, content, procedures, data usage and their rights and obligations. In cases where participants were unable to sign themselves, legal guardians signed on their behalf. To ensure data confidentiality, only basic demographic information was collected; personally identifiable information, such as names and ID numbers, was excluded. All data were anonymised using coded identifiers. Paper questionnaires were stored in locked cabinets, and electronic data were saved on password-protected computers or encrypted devices. Access to data was strictly restricted to authorised research personnel. Data were used solely for statistical analysis in this study. After the study, both paper and electronic data were stored securely in accordance with standard medical research data retention regulations to prevent leakage or misuse.

The development of the survey questionnaire was based on a comprehensive review of relevant literature from domestic and international sources. The preliminary survey and specific operational situations were considered to modify and delete inappropriate items, resulting in the final questionnaire.

The following describes the survey questionnaire:

#### 2.2.1 General information

Basic information about the patients, including gender, age, ethnicity, religious beliefs, place of residence, occupation, educational level and marital status, was collected.

#### 2.2.2 Investigation of the factors affecting emotional health (consisting of six dimensions and 30 items)

##### 2.2.2.1 Mindfulness practice

The Chinese version of the Freiburg Mindfulness Inventory, which was developed by Chen et al. (12), was used for assessment. It includes five items rated on a 5-point scale to evaluate the level of mindfulness. Higher scores indicate higher levels of mindfulness.

##### 2.2.2.2 Social support

The Medical Outcomes Study Social Support Survey, which is used for assessing social support, was modified based on the study by Sherbourne et al. (13). It consists of five items asking how often the respondents felt that someone could listen to their problems and offer advice, suggestions or information. Higher ratings indicate greater social support.

##### 2.2.2.3 Patient-centred communication

The Patient-Centered Communication Scale in Cancer Care was used to assess the level of PCC. The respondents were asked about the frequency of communication with their doctors, nurses and healthcare professionals. Higher ratings indicate a higher level of PCC (14).

##### 2.2.2.4 Beliefs about cancer

The Cancer Beliefs Scale was developed by Niederdeppe et al. (15). It includes four items, and the respondents indicate their level of agreement or disagreement with each statement. Higher ratings indicate stronger beliefs in overcoming cancer.

##### 2.2.2.5 Health self-efficacy

The team led by Gustafson has developed a series of psychological assessment tools for patients with cancer. The Health Self-Efficacy Scale (HSES) is one of the core scales among them and is used to measure an individual’s confidence in ‘managing their own health and coping with disease challenges’ (16). The health self-efficacy scale used in this study consists of three items and was adapted from the HSES. Higher ratings indicate stronger health self-efficacy.

##### 2.2.2.6 Emotional health

While developing HSES, the Gustafson team also designed an Emotional Health Scale (EHS) to assess the emotional state of patients with cancer (such as the reverse measurement of anxiety and depression, i.e. ‘emotional health level’). The description in the document, ‘four items’ and ‘the higher the score is, the stronger the emotional health’, exactly matches the dimensions and scoring rules of the short version of the EHS (with four items), and both are often jointly used for psychological assessment of patients with cancer (16).

### 2.3 Quality control

Before the survey, the investigators received unified training according to standardised protocols. During the survey, the respondents completed the questionnaires themselves. After the questionnaires were completed, the investigators checked the completeness and logical consistency of the responses. If there were any missing or problematic items, the respondents were asked to complete them immediately. Invalid questionnaires – defined as those with more than three missing items or those exhibiting obvious patterns in the responses – were excluded.

### 2.4 Statistical methods

The SPSS 26.0 software package was used. Data were shown as mean ± standard deviation ( $\bar{\text{x}}$ ± s). Spearman’s correlation analysis was used to analyse the correlations between mindfulness practice, social support, PCC, beliefs about cancer, health self-efficacy and emotional health. Factor analysis was conducted to assess construct validity by extracting common factors using principal component analysis. Confirmatory factor analysis was performed using the AMOS 21.0 software. Bootstrap analysis (2,000 bootstrap samples with a 95% confidence interval) was used to examine the mediating effects. Unless otherwise specified, a *p*-value of <0.05 was statistically significant. When x²/df <3, root mean square error of approximation (RMSEA) <0.08, goodness of fit index (GFI) >0.8, normed fit index (NFI) >0.8, comparative fit index (CFI) >0.9, incremental fit index (IFI) >0.9, parsimony-adjusted normed fit index (PNFI) >0.5, parsimony-adjusted comparative fit index (PCFI) >0.5 and parsimony-adjusted goodness of fit index (PGFI) >0.5, the model fits well with the data.

## 3 Results

### 3.1 Baseline characteristics of the study population

A total of 517 participants with gastrointestinal cancer were included, with a male-to-female participant ratio of 1.04:1 (264 vs 253). The patients’ age range was 31–83 years, with the highest proportion (47.3%) in the 51–60 years age group. The patients were distributed between urban (45.8%) and rural (44.1%) areas. Patients with a primary school education or below accounted for 41.2%, and those with a high/vocational school education accounted for 44.7%. In terms of marital status, the majority (approximately 88.0%) were married. Regarding per capita monthly income, the largest proportion (approximately 60.2%) fell within the range of 3,000–5,900 Yuan (CNY ≈ 2,070 USD). The distribution of disease stages was concentrated in stages II and III, accounting for 41.8% and 36.2%, respectively. Specific details are shown in **Table 1**.

**Table 1:** General demographic characteristics of the study population.

Variables	Categories	n	%
Gender	Male	264	51.1
	Female	253	48.9
Age (years)	≤40	36	7.0
	41-50	114	22.1
	51-60	245	47.3
	61-70	90	17.4
	≥71	32	6.2
Ethnicity	Han	506	97.9
	Minority	11	2.1
Religious Belief	Yes	32	6.2
	No	485	93.8
Place of Residence	Urban	237	45.8
	Town	52	10.1
	Rural	228	44.1
Occupation	Worker	106	20.5
	Farmer	104	20.1
	Businessperson	21	4.1
	Government and public institution employees	16	3.1
	Enterprise employees	91	17.6
	Self-employed	47	9.1
	Unemployed	32	6.2
	Other	100	19.3
Educational Level	Middle school or below	213	41.2
	High school/vocational school	231	44.7
	High school/vocational school	65	12.6
	Postgraduate or above	8	1.5
Marital Status	Unmarried	32	6.2
	Married	455	88.0
	Divorced	21	4.1
	Widowed	9	1.7
Per Capita Monthly Income (yuan)	<3000	168	32.5
	3000-5999	311	60.2
	6000-9999	32	6.2
	≥10000	6	1.2
Medical Insurance Type	Out-of-pocket	52	10.1
	Employees’ medical insurance	176	34.0
	Basic medical insurance for urban and rural residents	289	55.9
Disease Stage	Stage I	32	6.2
	Stage II	216	41.8
	Stage III	187	36.2
	Stage IV	82	15.9

### 3.2 Exploratory factor analysis results and reliability testing of patient emotional health

The overall Kaiser–Meyer–Olkin measure of the patient satisfaction questionnaire was 0.935, and the results of Bartlett’s test of sphericity were *χ*^2^ = 16,453.365 and *p* < 0.001. A principal component analysis was conducted, and a total of six common factors were extracted: mindfulness practice, social support, PCC, beliefs about cancer, health self-efficacy and emotional health. These factors accounted for a cumulative variance of 70.7%, indicating good scale validity. Reliability analysis results showed that Cronbach’s alpha coefficients for each common factor were all >0.7. The Cronbach’s alpha coefficients were as follows: mindfulness practice (*α* = 0.929), social support (*α* = 0.905), PCC (*α* = 0.887), beliefs about cancer (*α* = 0.828), health self-efficacy (*α* = 0.833) and emotional health (*α* = 0.915). These results suggest that the internal consistency of the questionnaire is high, and the reliability of its content is good (see **Table 2**).

**Table 2:** Cronbach’s alpha coefficients for reliability analysis.

Factors	Questionnaire Items	Cronbach’s α based on standardized items	Variance	Accumulated variance
Mindfulness Practice	X45-X50	0.929	15.282	15.282
Social Support	X6-X10	0.905	12.918	43.053
Patient-Centered Communication	X11-X17	0.887	14.853	30.135
Cancer Beliefs	X18-X21	0.828	9.101	62.982
Health Self-Efficacy	X35-X37	0.833	7.749	70.731
Emotional Health	X28-X31	0.915	10.828	3.881

### 3.3 Confirmatory factor analysis

**Figure 2:**
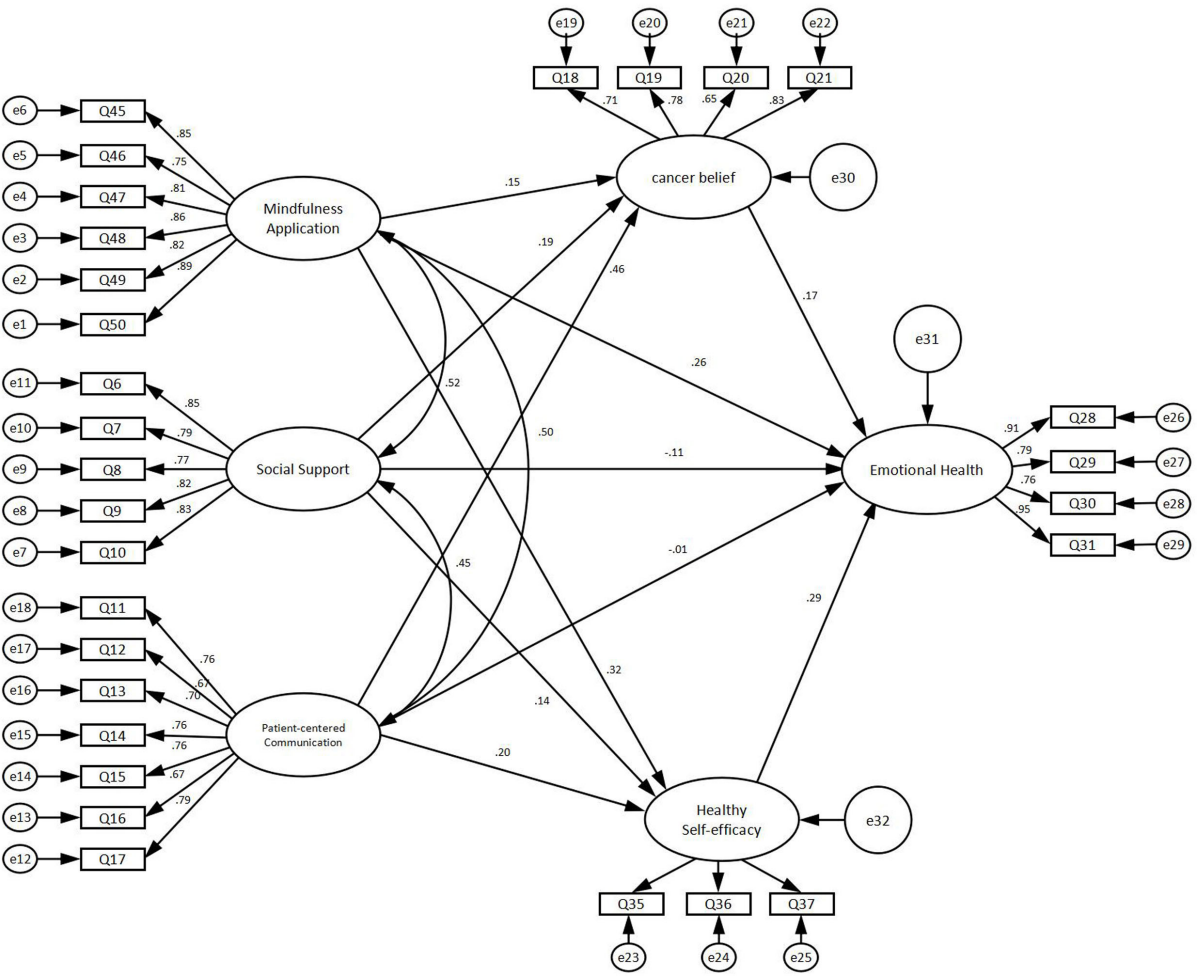
The model diagram for the confirmatory factor analysis.

The results of the confirmatory factor analysis showed that the x^2^/df value was 2.494, indicating a better model fit. The RMSEA was 0.054, indicating a good fit. The GFI was 0.894, NFI was 0.911, IFI was 0.945, CFI was 0.945, PNFI was 0.813, PCFI was 0.842 and PGFI was 0.744. These indicators were within the criteria, meaning that the validated factor analysis model developed was effective and fitted the collected data well (see **Table 3**). The model diagram for the confirmatory factor analysis can be found in **Figure 2**.

**Table 3:** Fit indices for confirmatory factor analysis model.

Goodness-of-fit Indices	Absolute Fit Indices			Incremental Fit Indices			Parsimonious Fit Indices		
	x²/df	RMSEA	GFI	NFI	IFI	CFI	PNFI	PCFI	PGFI
Fit Standards	<3	<0.08	>0.8	>0.8	>0.9	>0.9	>0.5	>0.5	>0.5
Model Parameters	2.494	0.054	0.894	0.911	0.945	0.945	0.813	0.842	0.744

The composite reliability values for each dimension were >0.7, indicating that all measurement items within each latent variable consistently explained that variable. This demonstrates good construct validity of the questionnaire. The average variance extracted (AVE) values were all >0.5, indicating good convergent validity of the scale (see **Table 4**).

**Table 4:** Composite reliability analysis results.

Latent Variable	CR Composite Reliability	AVE Average Variance Extracted
Mindfulness Practice	0.930	0.688
Social Support	0.905	0.657
Patient-Centered Communication	0.888	0.533
Cancer Beliefs	0.831	0.554
Health Self-Efficacy	0.833	0.625
Emotional Health	0.917	0.735

### 3.4 Discriminant validity and correlation analysis

The square root of the AVE for each latent variable was greater than the correlation coefficients between variables. This indicates that the variables in the scale have good convergence. Spearman’s correlation analysis results showed significant positive correlations (*p* < 0.01) between all pairs of variables. The strongest correlation was observed between beliefs about cancer and PCC, with a coefficient of 0.621. The weakest correlation was observed between health self-efficacy and social support, with a coefficient of 0.393 (see **Table 5**).

**Table 5:** Discriminant validity analysis results.

Items	Mindfulness Practice	Health Self-Efficacy	Emotional Health	Cancer Beliefs	Patient-Centered Communication	Social Support
Mindfulness Practice	0.829*					
Health Self-Efficacy	0.492	0.791*				
Emotional Health	0.538	0.527	0.857*			
Cancer Beliefs	0.479	0.430	0.463	0.744*		
Patient-Centered Communication	0.500	0.413	0.394	0.621	0.730*	
Social Support	0.526	0.393	0.440	0.480	0.451	0.811*

Bold values represents the square root of AVE, while the others represent the correlation coefficients between variables.

A multi-collinearity test was conducted using multiple linear regression analysis by including the independent and mediator variables in the regression model, with emotional health as the dependent variable. The results showed that all variance inflation factor values were <5, indicating the absence of severe multicollinearity (see **Table 6**).

**Table 6:** Correlation and multi-collinearity analysis results for each factor.

Items	Social Support	Patient-Centered Communication	Cancer Beliefs	Emotional Health	Health Self-Efficacy	Mindfulness Practice
Social Support Practice	1					
Patient-Centered Communication	0.409**	1				
Cancer Beliefs	0.423**	0.554**	1			
Emotional Health	0.444**	0.376**	0.422**	1		
Health Self-Efficacy	0.347**	0.363**	0.370**	0.486**	1	
Mindfulness Practice	0.478**	0.455**	0.431**	0.519**	0.442**	1
VIF	1.446	1.623	1.615	–	1.34	1.592

**represents *p* < 0.01.

### 3.5 Structural equation modelling analysis

**Figure 3:**
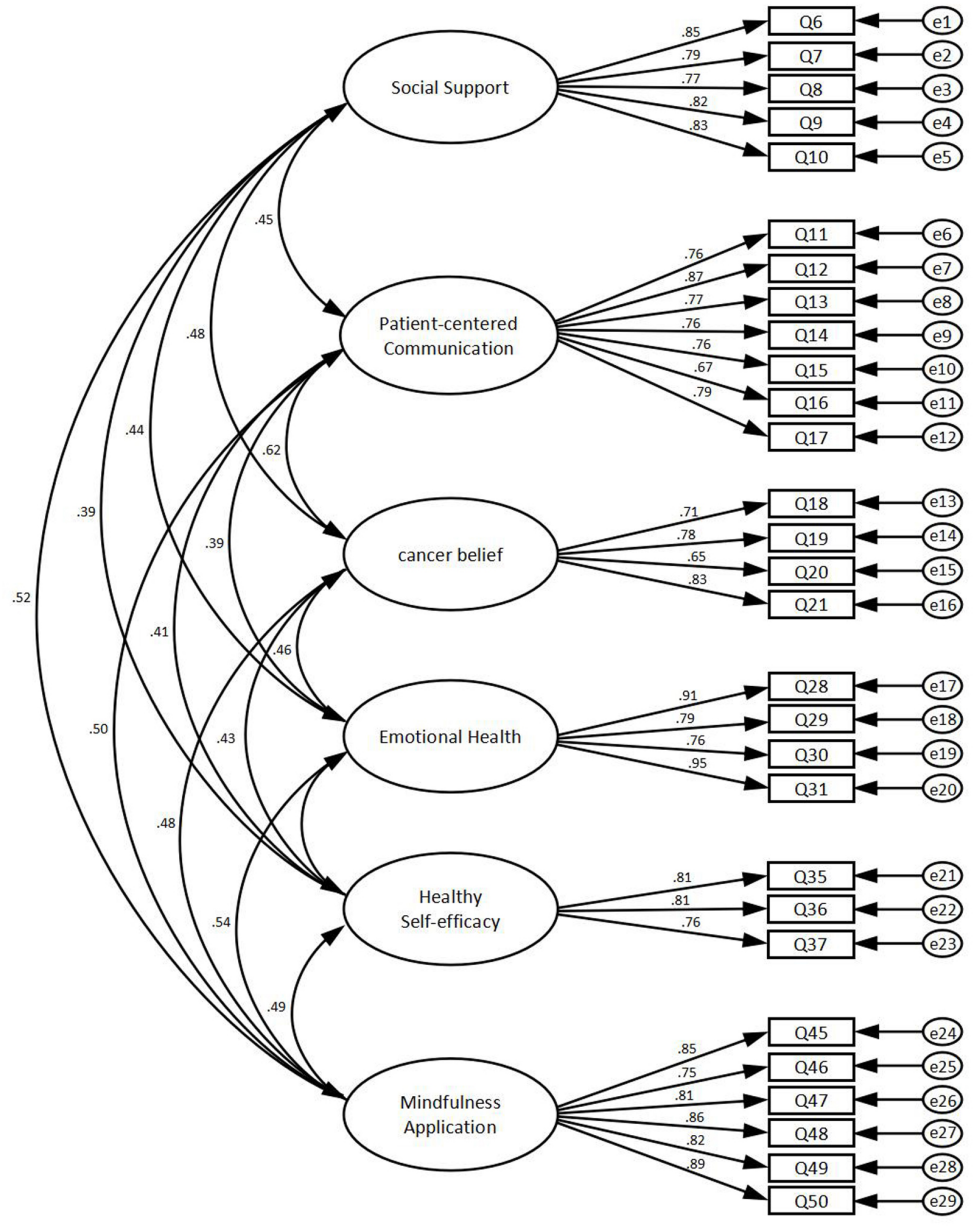
The structural equation model for the factors influencing emotional health in cancer patients.

A structural equation model was used to examine the factors influencing emotional health in patients with gastrointestinal cancer, with beliefs about cancer, health self-efficacy and emotional health, and mindfulness practice, social support and PCC considered as exogenous latent variables. The results of the structural equation modelling indicated a good fit for the model, with an x^2^/df value of 2.504 and an RMSEA of 0.054. The goodness-of-fit indices were as follows: GFI = 0.893, NFI = 0.911, IFI = 0.945, CFI = 0.944, PNFI = 0.814, PCFI = 0.844 and PGFI = 0.745. The above indices were all within the standard range, the structural equation model developed was effective and the model fit was good and acceptable (see **Table 7**, **Figure 3**).

**Table 7:** Fit indices for the structural equation model.

Fit Indices	Absolute Fit Indices			Incremental Fit Indices			Parsimonious Fit Indices		
	x²/df	RMSEA	GFI	NFI	IFI	CFI	PNFI	PCFI	PGFI
Fit Standards	<3	<0.08	>0.8	>0.8	>0.9	>0.9	>0.5	>0.5	>0.5
Model Parameters	2.504	0.054	0.893	0.911	0.945	0.944	0.814	0.844	0.745

X^2^ = 908.807, df = 363, *p* < 0.001.

Path analysis of structural equation models found that mindfulness practice (*p* < 0.001), social support (*p* = 0.022), beliefs about cancer (*p* = 0.003) and health self-efficacy (*p* < 0.001) all positively influenced emotional well-being. Mindfulness practice (*p* = 0.005), social support (*p* < 0.001) and PCC (*p* < 0.001) were found to influence beliefs about cancer positively. Mindfulness practice (*p* < 0.001), social support (*p* = 0.014) and PCC (*p* < 0.001) were also found to influence health self-efficacy positively (see **Table 8**).

**Table 8:** Path analysis results.

Hypothesized Paths			Estimate	S.E.	C.R.	*p*	Standardized Path Coefficients	Statistical Results
Mindfulness Practice	→	Emotional Health	0.293	0.059	4.955	<0.001	0.261	Supported
Social Support	→	Emotional Health	0.145	0.063	2.291	0.022	0.114	Supported
Patient-Centered Communication	→	Emotional Health	-0.017	0.073	-0.238	0.812	-0.013	Not Supported
Cancer Beliefs	→	Emotional Health	0.260	0.088	2.957	0.003	0.172	Supported
Health Self-Efficacy	→	Emotional Health	0.428	0.075	5.721	<0.001	0.290	Supported
Mindfulness Practice	→	Cancer Beliefs	0.109	0.039	2.81	0.005	0.147	Supported
Social Support	→	Cancer Beliefs	0.162	0.044	3.727	<0.001	0.193	Supported
Patient-Centered Communication	→	Cancer Beliefs	0.395	0.048	8.182	<0.001	0.462	Supported
Mindfulness Practice	→	Health Self-Efficacy	0.245	0.045	5.479	<0.001	0.322	Supported
Social Support	→	Health Self-Efficacy	0.118	0.048	2.449	0.014	0.137	Supported
Patient-Centered Communication	→	Health Self-Efficacy	0.174	0.049	3.55	<0.001	0.198	Supported

### 3.6 Mediating effects of beliefs about cancer and health self-efficacy

The results indicated the presence of mediation effects in the following pathways, utilising beliefs about cancer as a mediating variable: Path 1 [mindfulness practice − beliefs about cancer − emotional well-being] with an effect value of 0.025 (*p* = 0.002), Path 2 [social support − beliefs about cancer − emotional well-being], with an effect value of 0.033 (*p* = 0.003) and Path 3 [PCC − beliefs about cancer − emotional well-being], with an effect value of 0.079 (*p* = 0.003). Therefore, the hypothesis is supported.

**Figure 4:**
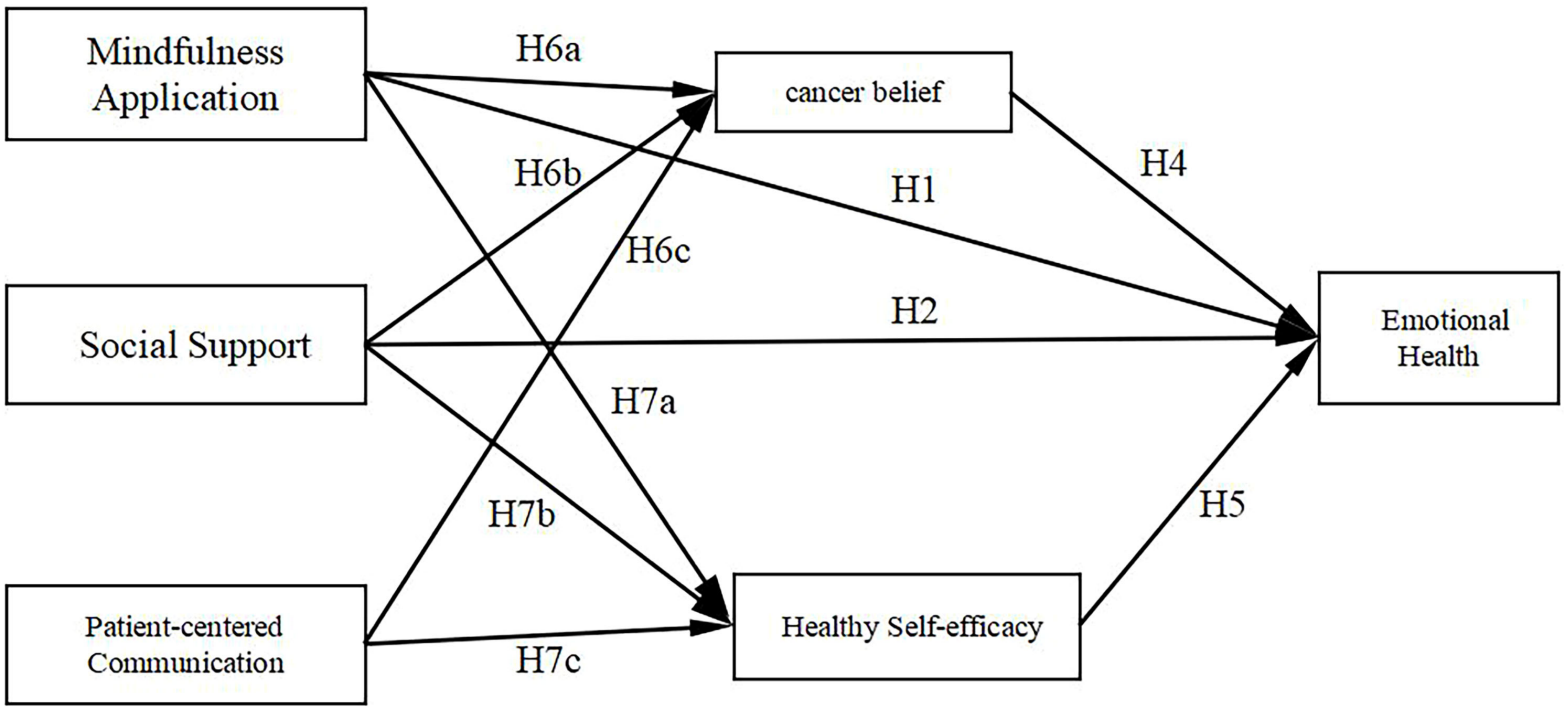
The confirmatory results of the research hypothesis.

The presence of mediation effects exists in the following pathways using health self-efficacy as a mediating variable: Path 4 [mindfulness practice − health self-efficacy − emotional well-being], with an effect value of 0.093 (*p* = 0.001), Path 5 [social support − health self-efficacy − emotional well-being], with an effect value of 0.040 (*p* = 0.019) and Path 6 [PCC − health self-efficacy − emotional well-being], with an effect value of 0.057 (*p* = 0.002). Therefore, the hypothesis is supported (see **Table 9**). The confirmatory results of the research hypothesis can be found in **Figure 4**.

**Table 9:** Mediating effects of cancer beliefs and health self-efficacy.

Mediating Effect Hypothesized Paths	Estimate	SE	95% Confidence Interval		*p*
			Lower Bound	Upper Bound	
1. Mindfulness Practice → Cancer Beliefs → Emotional Health	0.025	0.013	0.007	0.062	0.002
2. Social Support → Cancer Beliefs → Emotional Health	0.033	0.015	0.009	0.071	0.003
3. Patient-Centered Communication → Cancer Beliefs → Emotional Health	0.079	0.031	0.027	0.150	0.003
4. Mindfulness Practice → Health Self-Efficacy → Emotional Health	0.093	0.024	0.054	0.148	0.001
5. Social Support → Health Self-Efficacy → Emotional Health	0.040	0.018	0.007	0.080	0.019
6. Patient-Centered Communication → Health Self-Efficacy → Emotional Health	0.057	0.02	0.025	0.102	0.002

## 4 Discussion

The present study revealed two pivotal findings regarding emotional well-being in patients with gastrointestinal cancer from Northwest China. First, direct positive effects were confirmed for mindfulness practice, social support, adaptive beliefs about cancer and health self-efficacy on emotional well-being. Second, and more significantly, dual mediation pathways were identified; beliefs about cancer mediated 12.6%–31.7% of the total effects of mindfulness/social support/PCC, and health self-efficacy accounted for 23.2%–58.1% of these effects, with particularly strong mediation observed between mindfulness and well-being via self-efficacy (effect size, 0.09).

Mindfulness-based interventions are particularly helpful in coping with cancer diagnosis, treatment and survival (17). Mindfulness practice does not provide problem-solving assistance; however, it encourages individuals to turn towards difficult emotional experiences, embrace change and uncertainty and develop a gentle understanding of their bodies. This study found that mindfulness practice can positively influence the emotional health of patients with cancer. Tian (18) drew the same conclusion; he showed that interventions based on mindfulness may effectively alleviate anxiety, depression and fatigue in patients with lung cancer. In the context of Chinese culture, this ‘internal seeking’ adjustment method aligns perfectly with the ‘mental health preservation’ concept in traditional Chinese medicine, emphasising the achievement of physical and mental balance through regulating one’s own mindset and conforming to natural laws.

This study found that health self-efficacy has a positive effect on emotional health. Individuals with high self-efficacy have high expectations, approach situations rationally, are willing to face challenges, can control self-defeating thoughts and can apply wisdom and skills when needed (19). Research has found that improving self-efficacy can effectively reduce the occurrence of fear of gastrointestinal cancer in survivors (20). Furthermore, health self-efficacy mediates the effects of mindfulness practice, social support and PCC on emotional health.

Social support in patients with cancer mostly involves providing various forms of emotional and material comfort and care to help patients overcome their illness. This includes not only passive support received by patients with cancer but also the active seeking of support by patients and their ability to support others around them (21). This study demonstrated a correlation between social support and patient emotional health. Research has shown that when patients who survived cancer increase their communication with the outside world and actively seek help, they experience more happiness and higher self-esteem (22). The concept of ‘face’ in Chinese culture sometimes also affects patients’ willingness to seek external support (such as community resources, patient support groups) actively, fearing being regarded as ‘weak’ or ‘in need of help’. Therefore, social support in the Chinese context is complex and multifaceted, and it requires medical staff and family members to pay more careful attention to the methods and extent of providing support.

This study did not find a direct positive impact of PCC by healthcare professionals on emotional health. However, PCC can indirectly influence the emotional health of patients with cancer through beliefs about cancer and health self-management efficacy. Evidence suggests that improving communication can enhance patient satisfaction, improve treatment adherence and reduce negative emotions, ultimately producing a positive impact on physical health (23). Specifically, adaptive beliefs about cancer – such as viewing cancer as a manageable condition rather than a terminal illness – can enhance treatment adherence and reduce anxiety, thereby improving emotional outcomes (24). Health self-efficacy facilitates emotional well-being by promoting proactive coping strategies, such as seeking information and maintaining healthy behaviours, which reduce helplessness and strengthen emotional stability (25). Although no direct effect of PCC on emotional health was identified, it exerts a significant indirect influence by reshaping illness beliefs and enhancing self-efficacy through empathetic and clear communication (26). These findings underscore the importance of cognitive and self-regulatory pathways in psycho-oncological care and highlight the role of constructive patient-provider communication in fostering psychological adaptation.

This study found a positive effect of beliefs about cancer on patient emotional health. Additionally, beliefs about cancer mediate the effects of mindfulness practice, social support and PCC. Some studies have shown that patients who hold fatalistic beliefs about cancer deny the effectiveness of treatment and believe that the outcome of cancer is controlled by an external force that is unchangeable by human intervention (27). This further demonstrates that fatalistic beliefs about cancer can lead to low mood during the treatment process, poor adherence and subsequent negative effects on treatment outcomes.

According to the above results, in the care of patients with cancer, it can be helpful to increase mindfulness practice and develop appropriate mindfulness intervention programmes according to the patient’s condition in routine care. For example, targeted cancer diet and life care knowledge based on the type of cancer can reduce the patient’s concerns about their own disease care and uncertainty. To achieve this, practices such as mindfulness yoga, walking, breathing and meditation can be chosen according to the patient’s activity. Mindfulness practice may encounter some non-compliance; therefore, combined intervention modalities, such as face-to-face, telephone and WeChat communication, can be considered. Additionally, group intervention involving approximately 10 participants in each group could be used. Formal mindfulness exercises can be interspersed with some informal mindfulness exercises (28), such as seminars. Family members can be invited to discuss, analyse and explain troublesome issues, providing a channel for emotional output for patients and strengthening communication and encouragement between patients and family members, which can increase social support and improve self-management efficacy. In Chinese families, it is very natural and important to invite family members to participate in the psychological and rehabilitation process of the patient. Family members are not only the main caregivers but also the main source of emotional support for the patient. By organising family meetings or group activities, it is encouraged for family members to learn about the disease together, share their feelings and jointly develop coping strategies.

As the incidence of gastrointestinal cancer tumours has risen, people’s attention to such diseases has deepened. From the perspective of medical staff, nursing staff can build a scientific, reasonable and individualised psychological intervention programme based on research and individual differences, encourage patients to express their feelings, effectively guide their mood transfer and actively deal with problems. This helps to reduce patients’ psychological distress, improve their life satisfaction and maintain their best health status. From the patient level, the corresponding psychological counselling by medical staff can change the patient’s inner (wrong) view of gastrointestinal tumours, improve their level of benefit discovery after treatment and encourage them to better feel the meaning of life and the value of self-existence, thus reducing the psychological distress caused by various negative emotions. Therefore, the practice of this study will provide directional guidance for the nursing and treatment measures of patients with gastrointestinal tumours; this is of great significance for the improvement of patients’ mental health outcomes and quality of life.

However, this study has certain limitations. First, it did not consider the influence of general demographic and sociological factors on the emotional health of the study population, which may limit the explanatory power of the model. Second, the emotional health of patients with cancer is related to survival time and disease progression. However, this study only conducted a cross-sectional survey analysis and did not explore the changes in emotional health and influencing factors at different stages of the disease. Therefore, future research should consider conducting prospective cohort studies.

## 5 Conclusion

In summary, mindfulness practice, social support, beliefs about cancer and health self-efficacy have positive effects on the emotional health of patients with cancer. Beliefs about cancer and health self-efficacy mediate the effects of mindfulness practice, social support and PCC on emotional health. In the future, when designing health strategies and information for interventions and prevention measures targeting the emotional health of patients who survived cancer, these factors should be taken into consideration.

## Data Availability

The original contributions presented in the study are included in the article/supplementary material. Further inquiries can be directed to the corresponding author.

## References

[1] Arnold M Abnet CC Neale RE Vignat J Giovannucci EL McGlynn KA . Global burden of 5 major types of gastrointestinal cancer. Gastroenterology. (2020) 159:335–349.e15. doi: 10.1053/j.gastro.2020.02.068, PMID: 32247694 PMC8630546

[2] El Majzoub I Abunafeesa H Cheaito R Cheaito MA Elsayem AF . Management of altered mental status and delirium in cancer patients. Ann Palliat Med. (2019) 8:728–39. doi: 10.21037/apm.2019.09.14, PMID: 31735040

[3] Pearce M Garcia L Abbas A Strain T Schuch FB Golubic R . Association between physical activity and risk of depression: A systematic review and meta-analysis. JAMA Psychiatry. (2022) 79:550–9. doi: 10.1001/jamapsychiatry.2022.0609, PMID: 35416941 PMC9008579

[4] Walker J Mulick A Magill N Symeonides S Gourley C Burke K . Major depression and survival in people with cancer. Psychosom Med. (2021) 83:410–6. doi: 10.1097/PSY.0000000000000942, PMID: 33938501 PMC7614901

[5] Cramer H Lauche R Klose P Lange S Langhorst J Dobos GJ . Yoga for improving health-related quality of life, mental health and cancer-related symptoms in women diagnosed with breast cancer. Cochrane Database Syst Rev. (2017) 2017:CD010802. doi: 10.1002/14651858.CD010802.pub2, PMID: 28045199 PMC6465041

[6] Bekiros S Jahanshahi H Munoz-Pacheco JM . A new buffering theory of social support and psychological stress. PloS One. (2022) 17:e0275364. doi: 10.1371/journal.pone.0275364, PMID: 36223401 PMC9555651

[7] Zhang H Zhao Q Cao P Ren G . Resilience and quality of life: Exploring the mediator role of social support in patients with breast cancer. Med Sci Monit. (2017) 23:5969–79. doi: 10.12659/MSM.907730, PMID: 29248937 PMC5744469

[8] Wawrzynski SE Schaefer MR Schvaneveldt N Alderfer MA . Social support and siblings of children with cancer: A scoping review. Psychooncology. (2021) 30:1232–45. doi: 10.1002/pon.5689, PMID: 33851490 PMC8363579

[9] White LL Cohen MZ Berger AM Kupzyk KA Bierman PJ . Self-efficacy for management of symptoms and symptom distress in adults with cancer: An integrative review. Oncol Nurs Forum. (2019) 46:113–28. doi: 10.1188/19.ONF.113-128, PMID: 30547965

[10] Awick EA Phillips SM Lloyd GR McAuley E . Physical activity, self-efficacy and self-esteem in breast cancer survivors: a panel model. Psychooncology. (2017) 26:1625–31. doi: 10.1002/pon.4180, PMID: 27231845 PMC5555822

[11] Zhang D Lee EKP Mak ECW Ho CY Wong SYS . Mindfulness-based interventions: an overall review. Br Med Bull. (2021) 138:41–57. doi: 10.1093/bmb/ldab005, PMID: 33884400 PMC8083197

[12] Chen Y . Revision and psychometric evaluation of the Freiburg Mindfulness Inventory (FMI) among Chinese university students. Beijing: Beijing Normal University (2011).

[13] Sherbourne CD Stewart AL . The MOS social support survey. Soc Sci Med. (1991) 32:705–14. doi: 10.1016/0277-9536(91)90150-B, PMID: 2035047

[14] Epstein R Street RL . Patient-centered communication in cancer care: Promoting healing and reducing suffering. Bethesda, MD: National Cancer Institute (2007). NIH Publication No. 07-6225.

[15] Niederdeppe J Levy AG . Fatalistic beliefs about cancer prevention and three prevention behaviors. Cancer Epidemiol Biomarkers Prev. (2007) 16:998–1003. doi: 10.1158/1055-9965.EPI-06-0608, PMID: 17507628

[16] Gustafson DH McTavish FM Stengle W Ballard D Hawkins R Shaw BR . Use and impact of eHealth system by low-income women with breast cancer. J Health Commun. (2005) 10:195–218. doi: 10.1080/10810730500263257, PMID: 16377608

[17] Park S Sato Y Takita Y Tamura N Ninomiya A Kosugi T . Mindfulness-based cognitive therapy for psychological distress, fear of cancer recurrence, fatigue, spiritual well-being, and quality of life in patients with breast cancer—a randomized controlled trial. J Pain Symptom Manage. (2020) 60:381–9. doi: 10.1016/j.jpainsymman.2020.02.017, PMID: 32105790

[18] Li J Li C Puts M Wu YC Lyu MM Yuan B . Effectiveness of mindfulness-based interventions on anxiety, depression, and fatigue in people with lung cancer: A systematic review and meta-analysis. Int J Nurs Stud. (2023) 140:104447. doi: 10.1016/j.ijnurstu.2023.104447, PMID: 36796118

[19] Ceylan D Akan-Çelen FN Özkan S Aycan Z . Promoting adolescent health: health literacy, self-efficacy and internet use. Turk J Pediatr. (2022) 64:110–21. doi: 10.24953/turkjped.2021.1264, PMID: 35286037

[20] Ying X Wei Q Li D Gong W . Relationship between cancer fear of disease progression and cancer death anxiety, and cancer self-efficacy among lung cancer survivors. J Nurs Manage. (2022) 22:392–7. doi: 10.3969/j.issn.1671-315x.2022.06.003

[21] Deegan A Brennan C Gallagher P Lambert V Dunne S . Social support and childhood cancer survivors: A systematic review (2006-2022). Psychooncology. (2023) 32:819–33. doi: 10.1002/pon.6128, PMID: 36944590

[22] Zhang Y Ding Y Liu C Li J Wang Q Li Y . Relationships among perceived social support, family resilience, and caregiver burden in lung cancer families: A mediating model. Semin Oncol Nurs. (2023) 39:151356. doi: 10.1016/j.soncn.2022.151356, PMID: 36379816

[23] Gorman JR Standridge D Lyons KS Elliot DL Winters-Stone K Julian AK . Patient-centered communication between adolescent and young adult cancer survivors and their healthcare providers: Identifying research gaps with a scoping review. Patient Educ Couns. (2018) 101:185–94. doi: 10.1016/j.pec.2017.08.020, PMID: 28882546

[24] Peng M Zhang YP Wu Y Li R . Analysis of the influential factors of the emotional health of patients with cancer based on the structural equation model: the role of social media and emotional support. Support Care Cancer. (2023) 31:417. doi: 10.1007/s00520-023-07877-2, PMID: 37354259

[25] Ye JF Zheng S Ao SH Yan CD Lai Y Lai Z . How does patient-centered communication work? Trend analysis of mediation through cancer worry and health self-efficacy, 2011-2020. J Health Psychol. (2024) 29:1164–78. doi: 10.1177/13591053241228437, PMID: 38305168

[26] Tian X Zhou X Sun M Yu NX Peng Y Zheng X . The effectiveness of positive psychological interventions for patients with cancer: A systematic review and meta-analysis. J Clin Nurs. (2024) 33:3752–74. doi: 10.1111/jocn.17358, PMID: 38979929

[27] Francis DB Zelaya CM . Cancer fatalism and cancer information seeking among Black women: Examining the impact of Aretha Franklin’s death on cancer communication outcomes. J Cancer Educ. (2021) 36:763–8. doi: 10.1007/s13187-020-01701-9, PMID: 32020521

[28] Wang X . Effect of mindfulness-based stress reduction therapy on self-perceived burden and sleep in patients with colorectal cancer. Journal of Jilin University: Jilin University (2023). doi: 10.27162/d.cnki.gjlin.2022.005506

